# Protective effect of carbon dots derived from scrambled *Coptidis Rhizoma* against ulcerative colitis in mice

**DOI:** 10.3389/fmolb.2023.1253195

**Published:** 2023-08-30

**Authors:** Yanfang Mou, Xue Bai, Huagen Ma, Tingjie Li, Yafang Zhao, Tong Wu, Yue Zhang, Huihua Qu, Hui Kong, Xueqian Wang, Yan Zhao

**Affiliations:** ^1^ School of Traditional Chinese Medicine, Beijing University of Chinese Medicine, Beijing, China; ^2^ Henan University of Chinese Medicine, Zhengzhou, China; ^3^ School of Chinese Materia Medica, Beijing University of Chinese Medicine, Beijing, China; ^4^ School of Life Sciences, Beijing University of Chinese Medicine, Beijing, China; ^5^ Center of Scientific Experiment, Beijing University of Chinese Medicine, Beijing, China

**Keywords:** scrambled *Coptidis Rhizoma*, carbon dots, ulcerative colitis, intestinal tight junction proteins, inflammatory cytokines, oxidative stress, gut microbiota

## Abstract

**Introduction:** Ulcerative colitis (UC) is a chronic and progressive inflammatory disease of the intestines. The primary symptoms, such as bloody diarrhea, can result in weight loss and significantly diminish the patient’s quality of life. Despite considerable research endeavors, this disease remains incurable. The scrambled *Coptidis Rhizoma* (SCR) has a rich historical background in traditional Chinese medicine as a remedy for UC. Drawing from a wealth of substantial clinical practices, this study is focused on investigating the protective effects and underlying mechanisms of the active component of SCR, namely SCR-based carbon dots (SCR-CDs), in the treatment of UC.

**Methods:** SCR-CDs were extracted and isolated from the decoction of SCR, followed by a comprehensive characterization of their morphological structure and functional groups. Subsequently, we investigated the effects of SCR-CDs on parameters such as colonic length, disease activity index, and histopathological architecture using the dextran sulfate sodium (DSS)-induced colitis mice model. Furthermore, we delved into the assessment of key aspects, including the expression of intestinal tight junction (TJ) proteins, inflammatory cytokines, oxidative stress markers, and gut microbial composition, to unravel the intricate mechanisms underpinning their therapeutic effects.

**Results:** SCR-CDs displayed a consistent spherical morphology, featuring uniform dispersion and diameters ranging from 1.2 to 2.8 nm. These SCR-CDs also exhibited a diverse array of surface chemical functional groups. Importantly, the administration of SCR-CDs, particularly at higher dosage levels, exerted a noteworthy preventive influence on colonic shortening, elevation of the disease activity index and colonic tissue impairment caused by DSS. These observed effects may be closely associated with the hygroscopic capability and hemostatic bioactivity inherent to SCR-CDs. Concurrently, the application of SCR-CDs manifested an augmenting impact on the expression of intestinal TJ proteins, concomitantly leading to a significant reduction in inflammatory cell infiltration and amelioration of oxidative stress. Additionally, SCR-CDs treatment facilitated the restoration of perturbed gut microbial composition, potentially serving as a fundamental mechanism underlying their observed protective effects.

**Conclusion:** This study demonstrates the significant therapeutic potential of SCR-CDs in UC and provides elucidation on some of their mechanisms. Furthermore, these findings hold paramount importance in guiding innovative drug discovery for anti-UC agents.

## Graphical Abstract

**Figure:**
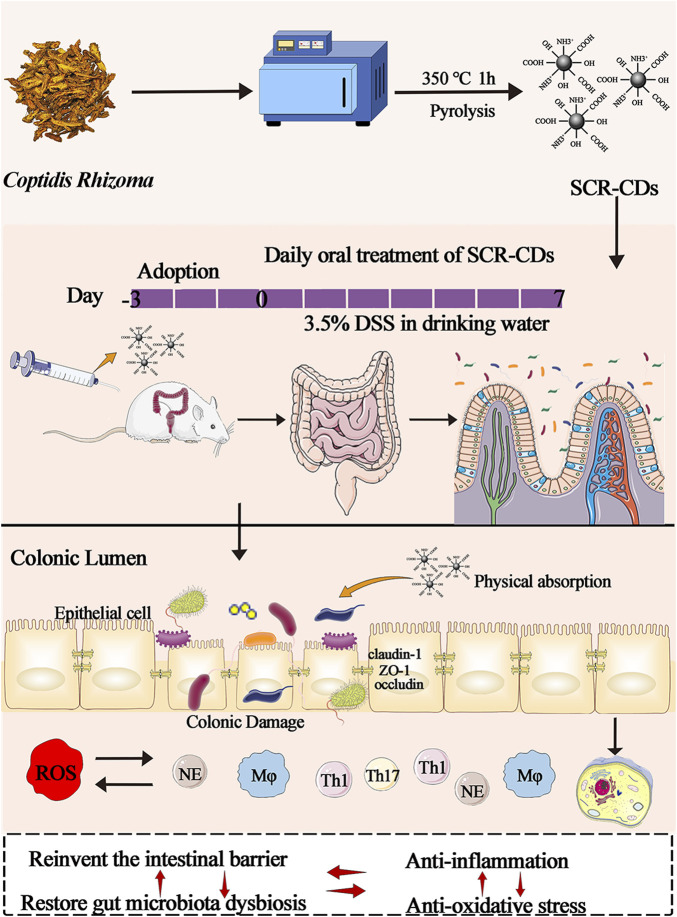
Schematic diagram of the synthesis of SCR-CDs and diagram of the experimental protocol.

## 1 Introduction

Ulcerative colitis (UC) is a nonspecific and immune-mediated inflammatory gastrointestinal disease, classified as one of the subtypes of inflammatory bowel disease (IBD). UC and Crohn’s disease (CD) are significant global health issues, impacting several million individuals, with their incidence and prevalence continuing to rise worldwide (Ashton et al., 2022; Huang J. G. et al., 2022). Moreover, these conditions impose a substantial economic burden on patients and severely affect their quality of life (Sachar, 2015). Furthermore, individuals with these conditions are at an elevated risk of developing colorectal cancer (Piovani et al., 2022). Currently, no curative treatment exists for UC, and the management primarily revolves around symptomatic pharmacotherapy (Ferretti et al., 2022). Despite the success of several monotherapies or combination therapies in achieving short-term and long-term remission and maintaining it, a substantial proportion of patients experience a loss of response to available treatments (Rowan et al., 2020). Even with the advent of new biological agents, the remission rate of UC continues to encounter a plateau in terms of achieving clinical targets. Consequently, there is an urgent demand to explore additional therapies that can complement conventional treatments.

The precise etiology of UC remains obscure and is likely to be multifactorial. In the initiation and progression of UC, disruption of the intestinal epithelial barrier increases the risk of bacterial invasion and translocation (Zou et al., 2020). The dysregulated immune response against commensal microflora was orchestrated by the mucosal immune system, leading to an alteration in the balance of the luminal microecological environmental homeostasis. Pathogens and translocated materials can induce the production of potent inflammatory cytokines and other chemical mediators in the colon (Zou et al., 2020). Subsequently, the excessive production of reactive oxygen species (ROS) and continuous oxidative stress can lead to tissue injury. Given that the gastrointestinal tract is the primary source of ROS, IBD has been characterized as an “oxyradical overload” disease (Chiba et al., 2012).

Fortunately, Traditional Chinese Medicine (TCM) has been providing medical assistance to patients for centuries and is often considered a potential treatment strategy. Among the various classic Chinese herbs available, *Coptidis Rhizoma* (CR) has been extensively utilized in clinical settings to alleviate gastrointestinal symptoms, including diarrhea, hematochezia, and stomachache. *Berberine* (BER) is a well-known main bioactive substance of CR. Numerous studies have reported that BER exhibits various beneficial properties, including anti-inflammatory effects (Zou et al., 2017), anti-oxidative activities (Liu et al., 2020), and anti-bacterial properties (Habtemariam, 2020). Despite its advantages, BER suffers from poor aqueous solubility, limited gastrointestinal tract absorption, and low bioavailability (approximately 5%). Consequently, high concentrations of BER must be administered to attain efficacy. This has also led to an elevated risk of adverse drug reactions, thereby restricting its clinical applicability (Dong et al., 2022).

In ancient Chinese medicine, physicians commonly employed the stir-frying method to reduce the toxicity of herbs, enhance their efficacy, and ensure their safe use in meeting clinical requirements. The application of SCR in gastrointestinal disorders has been established in healthcare practice for over two thousand years and continues to be widely accepted in China to this day. For now, the *Pharmacopoeia of the People’s Republic of China (2020)* remained recorded SCR processed by the stir-frying method. However, the pyrolysis inherent in the stir-frying process inevitably results in the degradation of specific BER components, leading to a reduction in the biological activity of CR. Therefore, it is reasonable to inquire whether SCR can continue to effectively ameliorate colitis and explore other biologically active ingredients, in addition to BER, that may exert its pharmacological effects.

Fortuitously, our team has previously uncovered that CDs obtained through the pyrolysis of Chinese medicine exhibit analgesic, antioxidant, antibacterial, and anti-inflammatory properties (Zhang M. et al., 2021; Zhang Y. et al., 2021). CDs are a novel class of zero-dimensional carbon nanomaterials with dimensions below 10 nm. Owing to their remarkable biological activity and ultra-low toxicity, CDs have found extensive applications in biomedicine. They serve as targeted nanocarriers, enhancing the efficacy of chemotherapeutic agents through their biocompatible properties. Additionally, CDs have shown promise in providing pharmacological therapy for the treatment of refractory diseases (Jin et al., 2022; Wang et al., 2022; Hsieh et al., 2023). Additionally, it is noteworthy to mention that semi-carbonized nanodots derived from charred *Atractylodes Macrocephala* demonstrated significant biological effects in maintaining the intestinal flora homeostasis of stress-induced gastric ulcer models (Lu et al., 2021). Hence, we postulate that the interaction with microorganisms could potentially serve as a mechanism underlying one aspect of the therapeutic effects of SCR-CDs derived from SCR in the treatment of UC.

Based on these clues, We investigated the protective effects of SCR-CDs against DSS-induced colitis mice model, and further elucidated the mechanisms of SCR-CDs in treating UC by examining the expression of intestinal TJ proteins, inflammatory cytokines, oxidative stress markers, and gut microbial composition. These findings provide further theoretical support for the application of SCR-CDs in the field of medical treatment.

## 2 Materials and methods

### 2.1 Materials

*Coptidis Rhizoma* (CR) was purchased from Beijing Qiancao Herbal Pieces Co., Ltd. (Beijing, China). Dialysis membranes (MWCO: 1000 Da) were purchased from Beijing Ruida Henghui Technology Development Co., Ltd. (Beijing, China). DSS (Molecular weight 36–50 kDa) was purchased from MP Biomedicals, Inc. (Irvine, CA, Uunited States). O-toluidine was purchased from the Beijing John Lunda Technology Development Co., Ltd. (Beijing, China). Haemocoagulase (HC) for injection was purchased from Jinzou Ahon Pharmaceutical Co., Ltd. (Liaoning, China). Enzyme-linked immunosorbent assay (ELISA) kits for mouse Tumor necrosis factor (TNF)-α, Interleukin (IL)-1β, IL-6, IL-17A, IL-22, IL-23, Granulocyte-macrophage colony-stimulating factor (GM-CSF), and Interferon (IFN)-γ were purchased from Jiangsu Kete Biotechnology Co., Ltd. (Jiangsu, China). Myeloperoxidase (MPO), Superoxide dismutase (SOD), Glutathione (GSH), Malondialdehyde (MDA), and Nitric oxide (NO) were purchased from the Nanjing Jiancheng Bioengineering Institute (Nanjing, China). Proteinase, phosphatase inhibitors and the BCA assay kit were purchased from Shanghai Beyotime Biotechnology Co., Ltd. (Shanghai, China).

### 2.2 Animals

Forty-eight male BALB/c mice and forty male Kunming mice were purchased from SiPeiFu Biotechnology Co., Ltd. (Beijing, China). The mice were reared in a clean-grade animal room (indoor temperature of 24°C ± 1°C and humidity of 50% ± 10%, under 12 h dark/light cycles). All animals were provided *ad libitum* access to food and water. All experimental procedures and animal care were performed according to the guidelines of the Care and Use of Laboratory Animals that were approved by the Ethics Committee of Animal Experimentation of Beijing University of Chinese Medicine.

### 2.3 Preparation of SCR-CDs

Initially, CR was loaded into a crucible with a lid and subjected to pyrolysis at 350 °C for 1 h in a muffle furnace (TL06112; Beijing ZhongKeAobo Technology Co., Ltd., Beijing, China). Subsequently, the SCR was decocted twice in deionized water (DW) at 100°C for 1 h each time. Following evaporation and concentration, the sample solution was filtered through a microporous membrane (Pore size 0.2 μm, Millipore). The solution was collected after 1 week in DW using a dialysis membrane to obtain the final purified sample containing SCR-CDs for later use. The schematic diagram of the experimental protocol for the preparation of SCR-CDs is exhibited in Supplementary Figure S1.

### 2.4 Characterization of SCR-CDs

The particle size and microscopic morphology of SCR-CDs were observed using a transmission electron microscope (TEM; Tecnai G2 20; FEI Company, Hillsboro, OR, United States). The atomic lattice spacing of SCR-CDs was uncovered utilizing a high-resolution TEM (JEN-1230; Japan Electron Optics Laboratory, Tokyo, Japan). The ultraviolet-visible (UV-vis) absorption spectra and photoluminescence characteristics of SCR-CDs were determined using a UV-vis spectrometer (CECIL, Cambridge, United Kingdom) and a fluorescence (FL) spectrophotometer (F-4500, Tokyo, Japan), respectively. Moreover, the functional groups and proportioning of chemical elements in SCR-CDs were characterized using Fourier transform infrared (FTIR) spectroscopy (Thermo Fisher, Fremont, CA, United States) and X-ray photoelectron spectroscopy (XPS; ESCALAB 250Xi, Thermo Fisher Scientific, Fremont, CA, United States), respectively. The zeta potential values and hydrodynamic diameter were determined using a Malvern Zetasizer Nano ZS90 (Malvern Instruments). The main components in the solutions of CR and SCR-CDs were identified using high-performance liquid chromatography (HPLC; Agilent 1260) with a ultraviolet detector at 265 nm.

### 2.5 Models of DSS-induced colitis model in mice and drug treatment

BALB/c mice were administered either regular DW or 3.5% DSS drinking water (with a new DSS solution provided every 2 days) following a 7 days acclimatization period. All animals were randomly divided into 6 groups, with 8 mice per group: 1) vehicle group (equal volume of DW); 2) DSS model group (DSS); 3) Sulfasalazine (SASP) administered group (500 mg/kg, DSS + SASP); 4) High-dose treatment group (0.96 mg/kg, DSS + H-SCR-CDs); 5) Medium-dose treatment group (0.48 mg/kg, DSS + M-SCR-CDs); 6) Low-dose treatment group (0.24 mg/kg, DSS + L-SCR-CDs). All animals received oral administration once daily for a duration of 7 days.

### 2.6 Disease activity index (DAI)

In this study, we investigated the protective effect of SCR-CDs against DSS-induced colitis in mice by evaluating DAI scores, a widely-used parameter for assessing the severity of colitis in animal models (Jeon et al., 2020). During the administration period, daily records were meticulously maintained for crucial parameters, including body weight, stool condition, as well as the presence of occult or gross bleeding in the mice. The DAI was calculated as the mean of the individual scores for the aforementioned parameters (Table 1), represented by the formula: DAI = (Weight loss + Stool condition + Occult or gross bleeding)/3.

**TABLE 1:** Parameters, grades, and scores of DAI.

Weight loss (%)	Stool condition	Occult or gross bleeding	Score
None	Normal	Negative	0
1–5		+	1
5–10	Loose stools	++	2
10–15		+++	3
˃15	Diarrhea	Gross bleeding	4

### 2.7 Sample collection and preparation

Upon conclusion of the experiment, the mice were expediently and humanely euthanized using cervical dislocation. Subsequently, a thorough rinse with phosphate-buffered saline (PBS) was performed, followed by precise measurement and division of the entire colon into two distinct segments. A designated section of the colon was allocated for the meticulous assessment of biochemical indicators. Concurrently, the remaining colon sections alongside pivotal major organs were meticulously immersed in a 4% neutral paraformaldehyde solution for fixation. Subsequent histological evaluation entailed staining with hematoxylin-eosin (H&E) to facilitate a comprehensive assessment of tissue morphology and structure.

### 2.8 Moisture absorption analysis

The moisture absorption of SCR-CDs was measured gravimetrically, following the methodology previously reported in the literature (Xu et al., 2020). During the sample preparation process, the material was first subjected to drying treatment by heating in an oven at 120 °C for 24 h until a constant weight was achieved. At the conclusion of the drying cycle, the spontaneously cooled sample was placed in a constant temperature and humidity chamber to achieve a stable condition (60% relative humidity at 25 °C room temperature) by absorbing water. During water vapor diffusion at a constant temperature, an analytical balance was used to weigh and record the real-time weight of the material. The percentage moisture absorption was calculated using Equation 1.

$$\mathrm{Percentage}\;moisture\;absorption=\frac{w_{t}-w_{i}}{w_{i}}\times 100\%$$ (1)

Where w_t_ and w_i_ are the weight of the sample at time “t” and the “initial weight” (g), respectively.

### 2.9 Hemostasis bioactivity evaluation

The tail-tip amputation and liver scratch models were established following previously published protocols (Sun et al., 2018; Zhang et al., 2018). Specifically, Kunming mice were randomly divided into 5 groups, with 8 mice per group: 1) vehicle group (equal volume of DW); 2) positive treatment group (0.67 Ku/kg, Haemocoagulase, HC); 3) SCR-CDs at different doses administered groups (0.96, 0.48, 0.24 mg/kg for the high-, medium-, low-dose groups). The animals were anesthetized, and a 1 cm segment was excised from the tip of the tail using small scissors. In addition, the trauma-hemorrhage model was established using a 1 mL syringe needle mimicking liver injury. The bleeding condition was monitored at 30 s intervals using filter paper, and the time was recorded until the Hemostatic endpoint was reached. Subsequently, all animals were humanely sacrificed by cervical dislocation.

### 2.10 Histological evaluation of colitis severity

The histological score, based on Cooper’s method, was determined by assessing inflammation severity, inflammation extent, and crypt damage (Cooper et al., 1993). In brief, the histopathological sections of the colon were scored based on the assessment of the following criteria: mucosa was normal and without inflammation, scored 0; mucosal goblet cell loss with mild inflammatory infiltration, scored 1; mucosal goblet cells were largely lost and moderate inflammatory infiltration was present, scored 2; mucosal crypt absence, extensive inflammatory infiltration, and mucosal edema thickening, scored 3; a large area of crypt loss and inflammatory infiltration of the submucosa, scored 4.

### 2.11 Immunofluorescence staining

Immunofluorescence was performed to determine the levels of ZO-1. For the immunofluorescence staining, the dewaxed sections were first blocked with 10% normal goat serum for 30 min at room temperature. Subsequently, the sections were incubated overnight with primary antibodies against ZO-1 (1:400; GB111981; Servicebio) at 4°C. Following this, the slices were washed and incubated with ZO-1-conjugated goat anti-rabbit secondary antibodies at 37°C for 50 min in the dark. After PBS washing, the sections were counterstained with DAPI for 10 min and sealed with 50% glycerol. Finally, the protein expression levels of ZO-1 were observed using a fluorescent microscope.

### 2.12 Western blot analysis

The protein concentration was quantified using a bicinchoninic acid (BCA) protein assay kit, and the samples were supplemented with loading buffer for western blot analysis. The proteins were separated by SDS-PAGE and subsequently transferred to PVDF membranes. After 1 h of blocking, the membranes were then incubated overnight at 4°C with specific primary antibodies, including anti-Claudin-1 (1:500; Ab15098; Abcam), anti-Occludin (1:1,000; Df7504; Affinity), ZO-1 (1:1000; Af5145; Affinity), and anti-β-actin (BM0627). Then the samples were incubated with the appropriate secondary antibody at room temperature for 1 h. Following the removal of the secondary antibody by washing, the protein bands were visualized using an ECL hypersensitive luminescent solution (Thermo Fisher, United States).

### 2.13 Determination of relevant biochemical indicators

The colon tissue was homogenized in normal saline to prepare 10% colon homogenate, which was subsequently centrifuged at 3,000 rpm for 10 min at 4°C to obtain the supernatant. The MPO activity and levels of inflammatory cytokines, including TNF-α, IL-1β, IL-6, IL-17A, IL-22, IL-23, GM-CSF, and IFN-γ, in the colon tissue were determined using commercial ELISA kits. Additionally, the oxidative stress indicators, including SOD, GSH, MDA, and NO levels, were measured following the manufacturer’s instructions.

### 2.14 16S rDNA gene high-throughput sequencing

Fecal samples and cecal contents were collected into sterile tubes and promptly stored at −80°C for preservation. Microbial genomic DNA was then extracted from the fecal samples using standard procedures. The final quantity and quality of DNA were assessed using Nanodrop spectrophotometry and 1.2% agarose gel electrophoresis, respectively. The V3-V4 hypervariable regions of the rRNA genes were amplified using a specific primer with a barcode. PCR amplification was performed using TransStart FastPfu DNA Polymerase (TransGen, Beijing, China). Fluorescence quantification of PCR amplification recovery products was performed using the Quant-iT PicoGreen dsDNA Assay Kit. Sequencing libraries were generated with TruSeq Nano DNA LT Library Prep Kit for Illumina. The library was sequenced by the MiSeq platform in the double-ended sequencing mode. Sequencing was completed by Shanghai Bioprofile Technology Company Ltd. (Shanghai, China). The statistical tests within the microbiome datasets were calculated using the Quantitative Insights Into Microbial Ecology (QIIME) pipeline.

### 2.15 Cell culture

GES-1 cells (human gastric epithelial cells) and RAW264.7 cells (mouse monocyte-macrophage leukemia cells) were cultured in 4.5 g/L D-glucose Dulbecco’s modified Eagle medium (DMEM) supplemented with 20% fetal bovine serum (FBS) and 1% penicillin-streptomycin (PS) solution at 37°C with 5% CO_2_.

### 2.16 Cell viability assay

GES-1 cells and RAW264.7 cells were seeded in a 96-well plate with 100 μL of culture media. The culture media were then replaced with different concentrations of SCR-CDs (SCR-CDs powder after freeze-drying, diluted with the culture medium), and the cells were incubated for an additional 24 h after they had fully adhered. Subsequently, the plates were incubated with 10% cells counting kit-8 solution (CCK-8) for 1 h at 37°C with 5% CO_2_. The optical density (OD) was measured at 450 nm using a microplate reader.

### 2.17 Blood biochemical indicators

At the conclusion of the experiment, blood was collected from the animals following a 12 h fasting period. The animals were swiftly euthanized by cervical dislocation, and the serum was subsequently separated through centrifugation for further biochemical analysis. The hematological parameters, including ALT (Alanine aminotransferase), AST (Aspartate aminotransferase), BUN (Blood urea nitrogen), CRE (Blood creatinine) were obtained using a Beckman Coulter CX4 Pro automatic biochemical analyzer (Beckman Coulter, Brea, CA, United States).

### 2.18 Statistical analysis

Statistical analysis was conducted using IBM SPSS Statistics software (version 20). The comparison of statistical differences between the two groups was performed using one-way analysis of variance (ANOVA), followed by LSD post-hoc tests. The results are presented as mean ± SD (standard deviations).

## 3 Results and discussion

### 3.1 Synthesis and characterization of SCR-CDs

**FIGURE 1:**
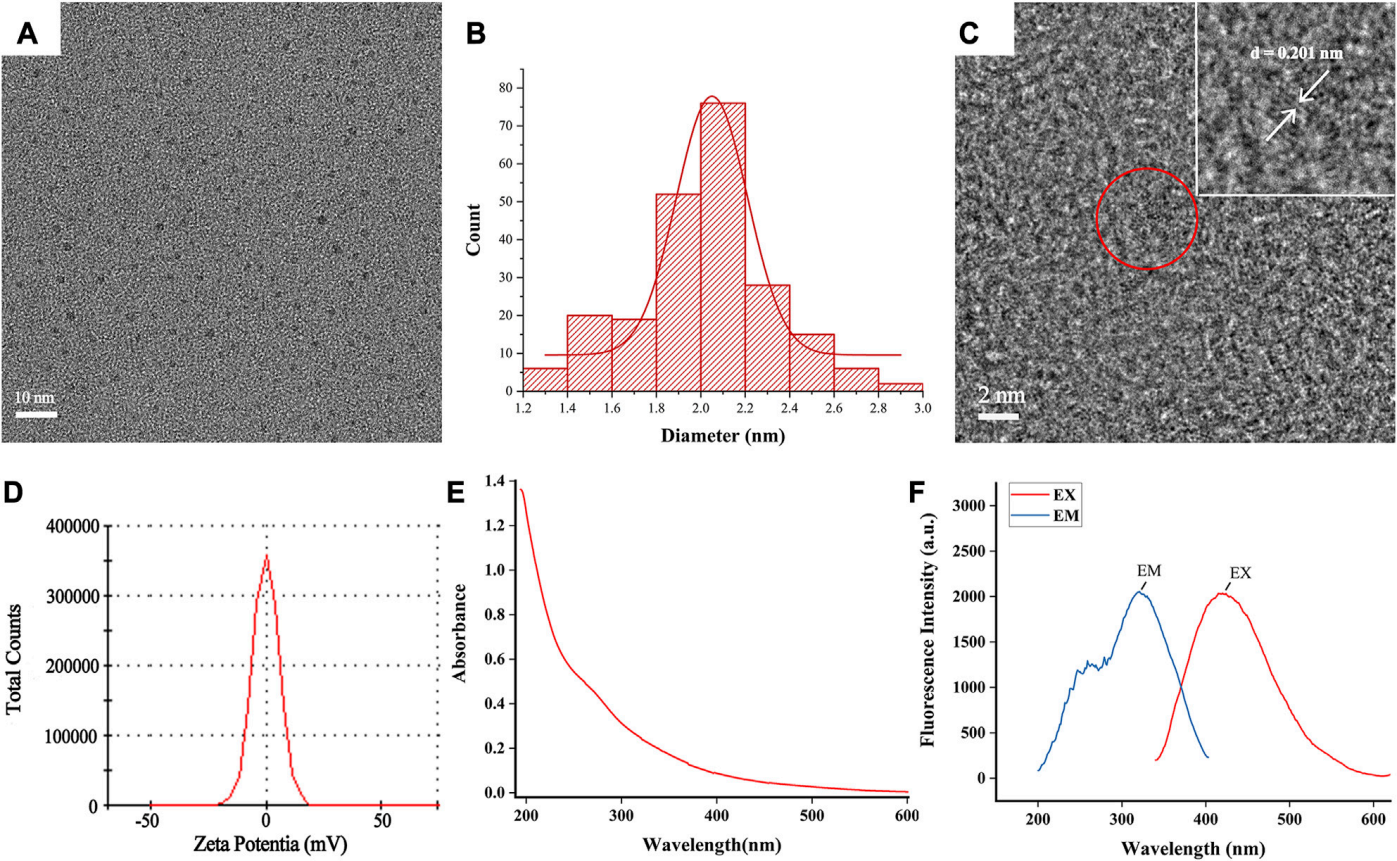
Ultrastructure characterization and optical properties of SCR-CDs. **(A)** Transmission electron microscopy (TEM) images of SCR-CDs displaying ultra-small particles. **(B)** Particle size distribution histogram of SCR-CDs. **(C)** High-resolution TEM image of individual SCR-CDs. **(D)** Zeta potential. **(E)** Ultraviolet–visible spectrum. **(F)** Fluorescence spectra.

TCM possesses the unique advantage of being multi-targeted, safe, and effective in the treatment of UC (Liu et al., 2022). SCR has long been recognized as one of the vital traditional herbs extensively employed in the treatment of gastrointestinal diseases in China. Its application has been documented in ancient medical literature, and its efficacy has been substantiated through numerous clinical practices. In this study, we utilized a muffle furnace with customized time and temperature parameters to control the degree of pyrolysis during the preparation of SCR. We successfully synthesized SCR-CDs through a simple and eco-friendly calcination method at 350°C for 1 h, avoiding the complexity and instability often associated with other synthesis methods (Supplementary Figure S1). To investigate the ultrastructure and morphology of SCR-CDs, we utilized TEM measurements. The obtained results revealed that SCR-CDs exhibited a roughly spherical structure (Figure 1A). The diameter of SCR-CDs ranged from 1.2 nm to 2.8 nm, with an average particle size of 2.0 nm (Figure 1B). High-resolution TEM enables the characterization of sample morphology with exceptional precision and provides detailed insights into microscopic physical properties. In the Figure 1C, two solid lines delineate distinct crystallographic planes of the matrix. Notably, the core of the nanowire exhibits well-resolved lattice fringes, revealing a lattice spacing of d = 0.201 nm. Moreover, SCR-CDs exhibited a positive zeta potential of + 0.0808 mV (Figure 1D). The observation of the Tyndall phenomenon, as depicted in Supplementary Figure S2, further supports the notion that SCR-CDs form a stable colloidal system in water. Furthermore, the SCR-CDs exhibited ultraviolet absorption properties, as confirmed by UV-vis spectral analysis (Figure 1E). Additionally, fluorescence characterization of the SCR-CDs revealed that the optimal excitation and maximum emission wavelengths were 321 nm and 420 nm, respectively (Figure 1F).

**FIGURE 2:**
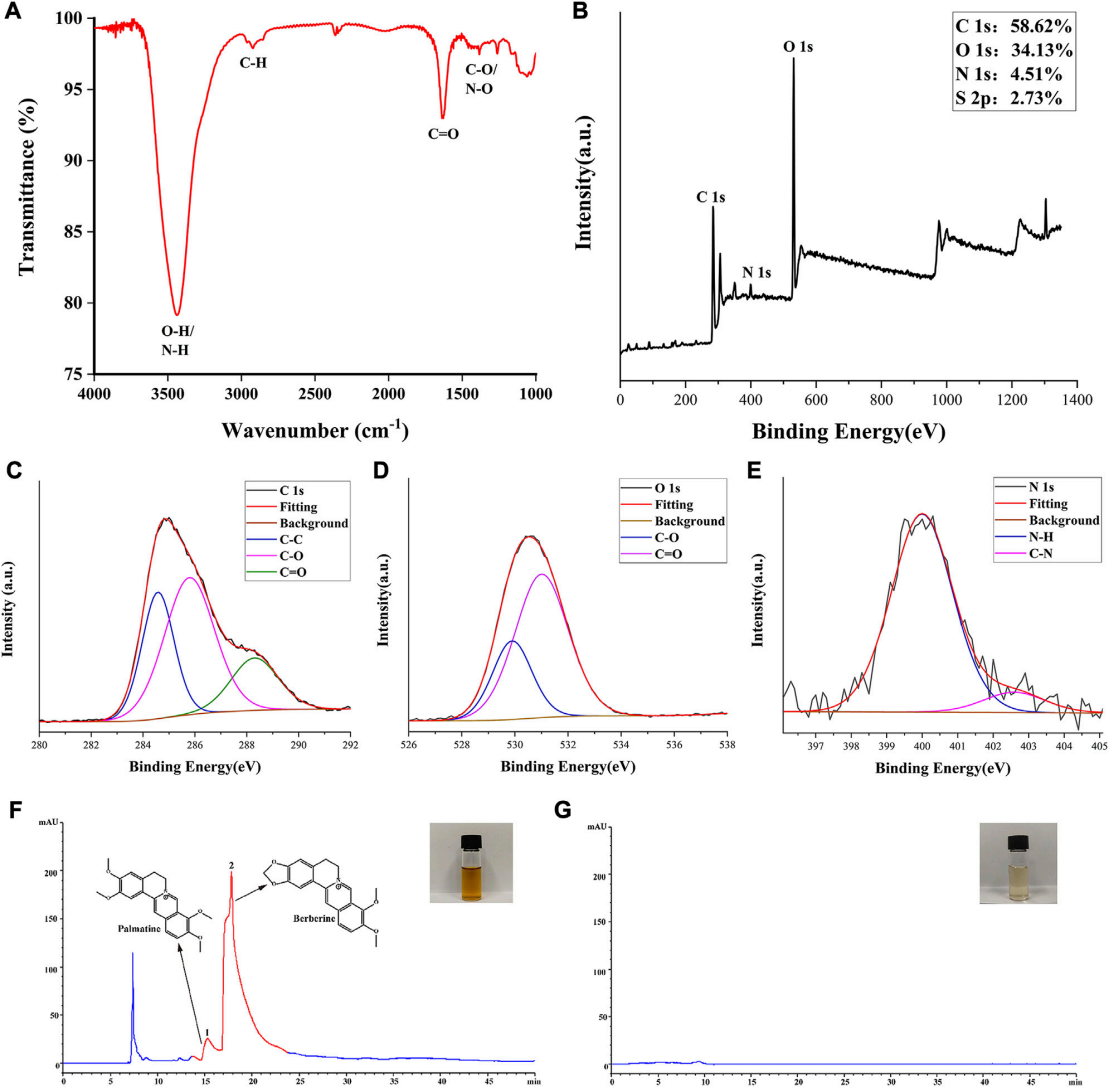
Elemental composition, functional group and main constituents of SCR-CDs. **(A)** Fourier transform infrared spectrum of SCR-CDs. **(B)** Full survey spectrum of X-ray photoelectron spectroscopy (XPS). High-resolution survey spectra of **(C)** C1s, **(D)** O1s and **(E)** N1s by XPS. The high-performance liquid chromatography fingerprint spectra of CR **(F)** and SCR-CDs **(G)**.

The FTIR spectra were analyzed to discern the surface functional groups of the SCR-CDs (Figure 2A). FTIR analysis depicted the peak at 3,438 cm^-1^ that belonged to the overlapping peaks of -OH (Ţucureanu et al., 2016) and -NH (Jafari et al., 2022) in the sample. A peak at 2,922 cm^-1^ was observed, which was assigned to the stretching C-H mode (Qambrani et al., 2022). Additionally, the FTIR spectrum showed the presence of stretching C=O modes at 1636 cm^-1^ (Feng et al., 2022). Furthermore, a peak at 1384 cm^-1^ was identified, corresponding to the C-O and N-O stretching in the carbohydrate, which may have resulted from dehydration during pyrolysis (Jalali et al., 2021). Based on these characterizations, it can be inferred that SCR-CDs with nanoscale dimensions exhibit optical properties of photoluminescence and fluorescence. To further assess the surface chemical composition and elemental status, we utilized XPS techniques to characterize the surface chemistry. Figure 2B showed the wide-range XPS spectra of the sample. The elemental composition of C, O, and N were determined to be 58.62%, 34.13%, and 4.51%, respectively. Three peaks were fitted to the C 1s spectrum of the region, further assigned to C-C (284.57 eV) (Zhao et al., 2022), C-O (285.79 eV) (Luo et al., 2022), and C=O (288.30 eV) (Noli et al., 2022) (Figure 2C). Due to the overlapping of peaks in the O 1s spectrum, we fitted two peaks corresponding to C-O (529.8 eV) (Zhu et al., 2022) and C=O (531.0 eV) (Shu et al., 2022) (Figure 2D). For the N 1s spectra, two peaks at 400.00 eV and 402.59 eV were observed, which could be attributed to the N-H (Shu et al., 2022) and C-N (Shaikh et al., 2022) bonds, respectively (Figure 2E). The accuracy of surface element identification was enhanced by considering the consistent information from both XPS and FTIR spectra.

The HPLC results revealed the presence of several compounds in the CR solution, including palmatine and BER (Figure 2F). However, as depicted in Figure 2G, the characteristic peak of the major component from CR was absent in the aqueous SCR-CDs after pyrolysis, isolation, and purification. This finding provides further evidence that active small molecule compounds were not present in SCR-CDs.

### 3.2 SCR-CDs alleviated the clinical symptoms in DSS-induced mice

**FIGURE 3:**
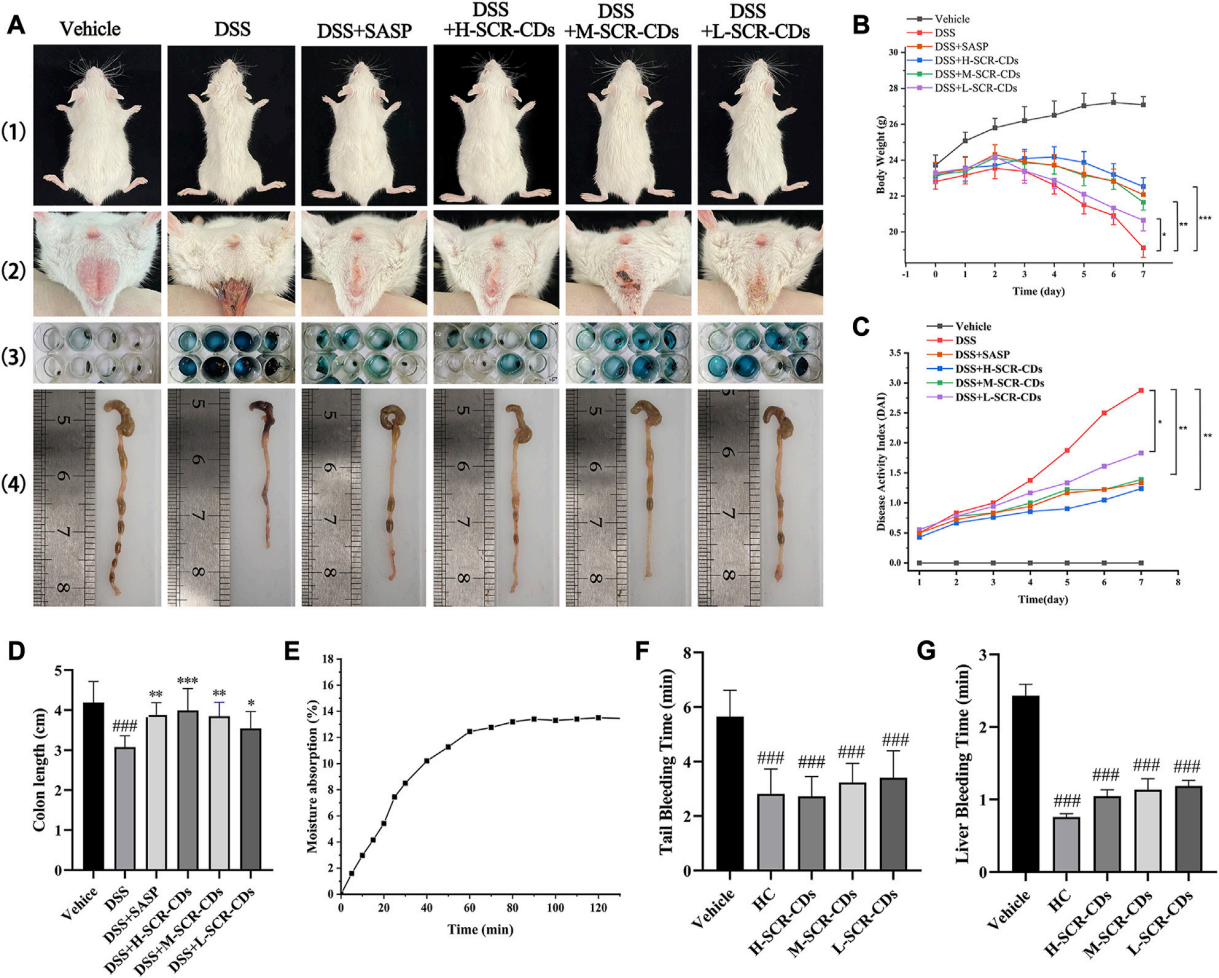
Effects of SCR-CDs on clinical symptoms in DSS-induced colitis mice and evaluation of SCR-CDs’ hygroscopic and hemostatic bioactivities. **(A)** 1) Presentation of gross body size of animals; 2) Soiled perianal region and accumulation of excreta at the anal; 3) Representative photograph of fecal occult blood test (blue corresponds to positive); 4) Macroscopic appearances of the colon. **(B)** Body weight changes in each group of mice. **(C)** DAI in each group of mice. **(D)** Histogram of colon length. **(E)** Moisture absorption analysis of SCR-CDs. **(F)** Tail amputation and **(G)** liver scratch models were treated with DW, HC, and different concentrations of SCR-CDs. Data are expressed as mean ± SD (*n* = 8). ^###^ *p* ˂ 0.001 versus vehicle group; *p ˂ 0.05, **p ˂ 0.01 and ***p ˂ 0.001 versus DSS group.

The effectiveness of SCR-CDs on colitis symptoms in mice subjected to 3.5% DSS in drinking water for 7 days post-induction were illustrated in Figure 3A. In addition to the body weight of normal mice increasing over time, DSS-exposed mice experienced marked weight loss due to colonic inflammation. Remarkably, varying degrees of body weight recovery were observed in the different doses of the SCR-CDs-treated group (Figure 3B).

Furthermore, a progressive increase in DAI scores was not only associated with the severity of weight loss but also related to the incidence of diarrhea and rectal bleeding. Based on these, DAI was a reliable indicator to evaluate colon inflammation and damage. The DSS group exhibited a higher likelihood of hematochezia and colon shortening compared to the vehicle group. In each of the other groups, the DAI increased with time in comparison to the vehicle group. However, the group treated with DSS individually showed the most significant increase in DAI (Figure 3C). Interestingly, the treatment with SCR-CDs significantly reduced the DAI and ameliorated colon length shortening (Figure 3D) in the experimental colitis mice. Notably, mice in the high-dose SCR-CDs group exhibited greater improvement compared to the other administration groups.

The moisture absorption curves of SCR-CDs demonstrated an extremely rapid initial rate of moisture uptake (Figure 3E). As water vapor gradually penetrated the sample, its mass increased with time, reaching moisture sorption equilibrium after 1 h. The hygroscopicity of SCR-CDs can be attributed to the presence of hydrophilic groups, such as -NH_2_, which readily form hydrogen bonds with water molecules. This hypothesis was further confirmed by the XPS results. In contrast to BER, SCR-CDs exhibit enhanced absorptive capacity and contribute to the alleviation of diarrhea symptoms. The physical property similar to activated carbons was also related to other medicinal effects in disease models (Hu et al., 2021; Lu et al., 2021; Luo et al., 2021).

Interestingly, we made a further discovery that SCR-CDs shortened the tail bleeding time (Figure 3F) and liver bleeding time (Figure 3G) in untreated mice, indicating that SCR-CDs enhanced the physiological hemostasis process. This effect could be attributed to formation of a protein corona with serum proteins after CDs were absorbed into the bloodstream, which might further impact the coagulation pathway (Fleischer and Payne, 2014). The symptomatic resolution is a primary clinical endpoint in treating UC with complicated pathogenesis at present. Consequently, the hemostasis effect of SCR-CDs may show therapeutic potential in relieving the clinical symptom of UC, especially bloody stool.

In this section, the nanosized SCR-CDs after purification of SCR had hygroscopic and hemostatic bioactivities that BER did not. It is noteworthy that positively charged SCR-CDs are expected to be readily taken up, given that epithelial cells typically display a net negative surface charge (Bannunah et al., 2014). Thus, SCR-CDs were administered in much lower doses than BER, establishing a prerequisite for the safety of SCR-CDs upon entry into the biological environment. These effect of SCR-CDs served as a motivation to further explore their underlying mechanisms in the treatment of different subtypes of IBD.

### 3.3 SCR-CDs ameliorated the infiltration of inflammatory cells and restored the mucosal barrier in DSS-induced mice

**FIGURE 4:**
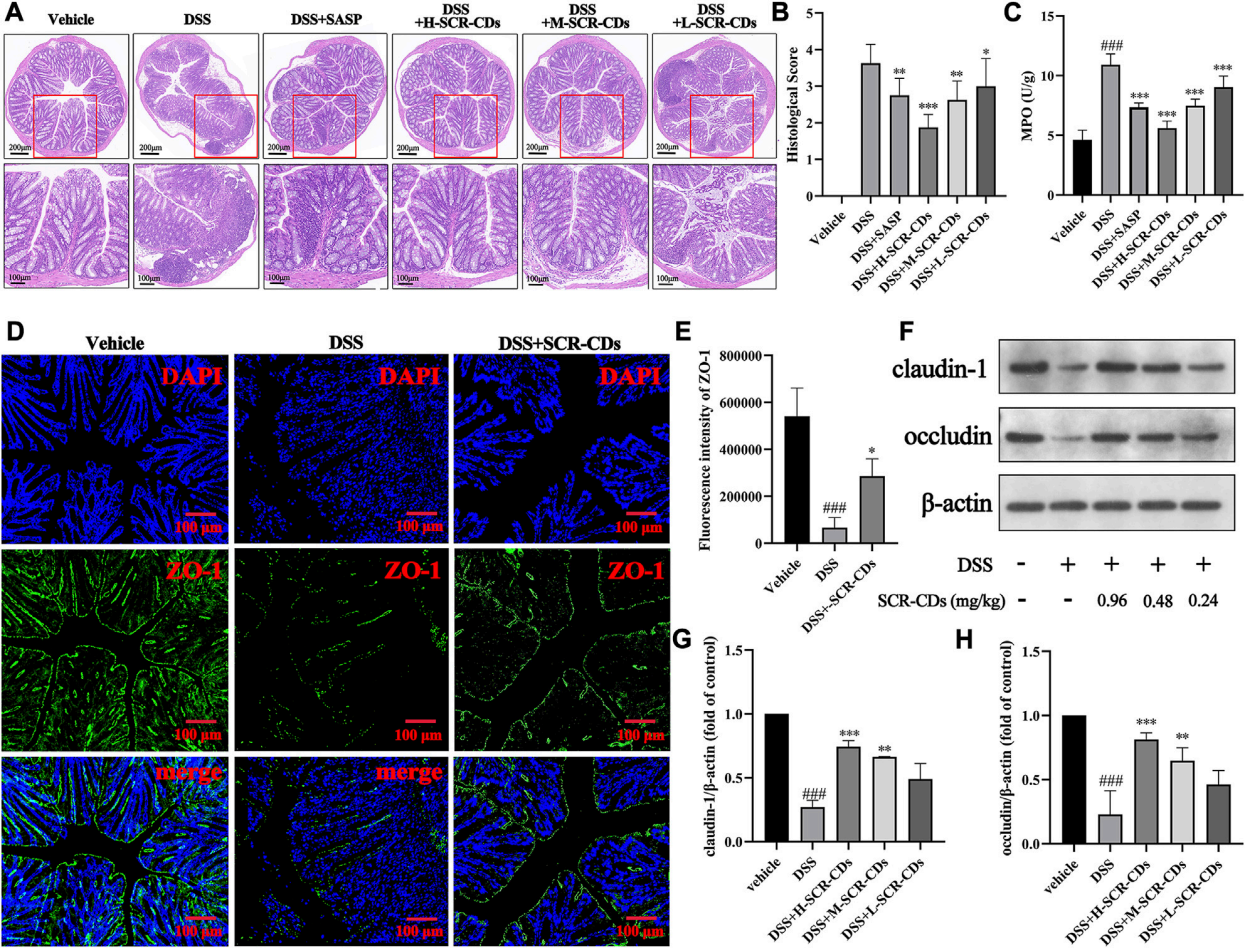
The effects of SCR-CDs on colonic inflammatory injury and the expression of intestinal tight junction proteins in DSS-induced mice. **(A)** Representative light microscopy image of H&E staining colon tissue sections (×100 magnification, Scar bar = 200 μm; ×200 magnification, Scar bar = 100 μm). **(B)** Statistical analysis of the pathological histological scores. **(C)** MPO activity in the colon homogenates. **(D–E)** Immunofluorescence for ZO-1 in colonic tissue and its quantified results (×100 magnification, Scar bar = 200 μm). **(F)** Immunoblot for claudin-1 and occludin in colonic tissue. **(G–H)** Immunoblot analysis of the protein density of claudin-1 and occludin in colonic tissue. Data are expressed as mean ± SD (*n* = 8). ^###^ *p* ˂ 0.001 versus vehicle group; *p ˂ 0.05, **p ˂ 0.01 and ***p ˂ 0.001 versus DSS group.

To quantify the extent of colonic inflammatory injury in each group of mice, we observed H&E staining sections (Figure 4A) and calculated a pathological histological score (Figure 4B). In normal mice, colonic sections exhibited a clear texture of the wrinkled wall, smooth mucosa, intact epithelium, absence of inflammatory cell infiltration, and no signs of congestion, edema, or ulcers in the submucosal layer. However, in DSS-treated mice, we observed focal epithelial necrosis, exfoliation of colonocytes and goblet cells, crypt disappearance, and submucosal edema with abundant neutrophil infiltration. Remarkably, the administration of SCR-CDs exhibited significant protective effects against DSS-induced colon damage. Microscopic manifestations showed the regeneration of the mucosal epithelium, restoration of tissue integrity, increased presence of colonocytes and goblet cells, reduced neutrophil infiltration, and efficient alleviation of congestion in the submucosa. Based on the inflammatory cell infiltration observed in the pathological sections, we further assessed neutrophil infiltration in the intestinal tissue through measurement of MPO activity. Neutrophil infiltration is the marker of intestinal inflammation in experimental IBD (Wu et al., 2023). MPO, as its characteristic enzyme, can induce damage to intestinal mucosal cells, inciting inflammatory responses, and exhibits a direct positive correlation with the severity of the disease (Wang et al., 2023). As depicted in Figure 4C, the MPO activity significantly increased to 10.91 U/g following DSS induction compared to the vehicle group (4.62 U/g). In contrast, the high-dose SCR-CDs treated group exhibited a modest elevation in MPO activity, reaching only 5.60 U/g. Based on the aforementioned results, SCR-CDs exhibited protective effects against colitis, including reduced mucosal erosions and decreased inflammatory infiltrations.

The tightly intact intestinal barrier is essential for segregating the appropriate microbial population and maintaining the homeostasis of multicellular organisms (Wells et al., 2022). The TJ proteins play a crucial role in preventing or regulating the invasion by microorganisms and are considered the primary structural component controlling paracellular permeability (Zuo et al., 2020). Numerous studies have indicated that upon entering a biological environment, especially in the presence of proteins, the surface of CDs rapidly gets coated with a layer of biomolecules (Nel et al., 2009; Mahmoudi et al., 2011). The protein corona on the surface of CDs undergoes dynamic changes over time as different proteins compete and replace each other. Upon entry into the colonic environment, the positively charged SCR-CDs might adhere to the intestinal epithelial cells (IECs) and become coated with a protein corona containing TJ proteins, which may contribute to the remodeling of the intestinal barrier. To validate this hypothesis, we performed immunofluorescence staining and western blot analysis to detect the expression of intestinal TJ proteins, and the results obtained were consistent with previous research findings (Ye et al., 2022). In Figures 4D–H, Supplementary Figure S3, it was observed that the protein levels of ZO-1, claudin-1, and occludin were significantly downregulated after the DSS induction (*p* < 0.05). The decrease in the expression of these three intestinal TJ proteins and the compromised integrity of the gut barrier in DSS-induced mice were consistent with previous studies (Li et al., 2022). Remarkably, following the administration of SCR-CDs, a notable increase in the levels of intestinal TJ proteins was observed. Among the groups treated with SCR-CDs, the high-dose group exhibited the most pronounced effect in enhancing the positive expression of these metrics. Taken together, these findings indicate that SCR-CDs play a substantial role in maintaining the integrity of the epithelial barrier and augmenting the expression of intestinal TJ proteins.

### 3.4 SCR-CDs effectively regulated the intestinal microecological environment in DSS-induced mice

**FIGURE 5:**
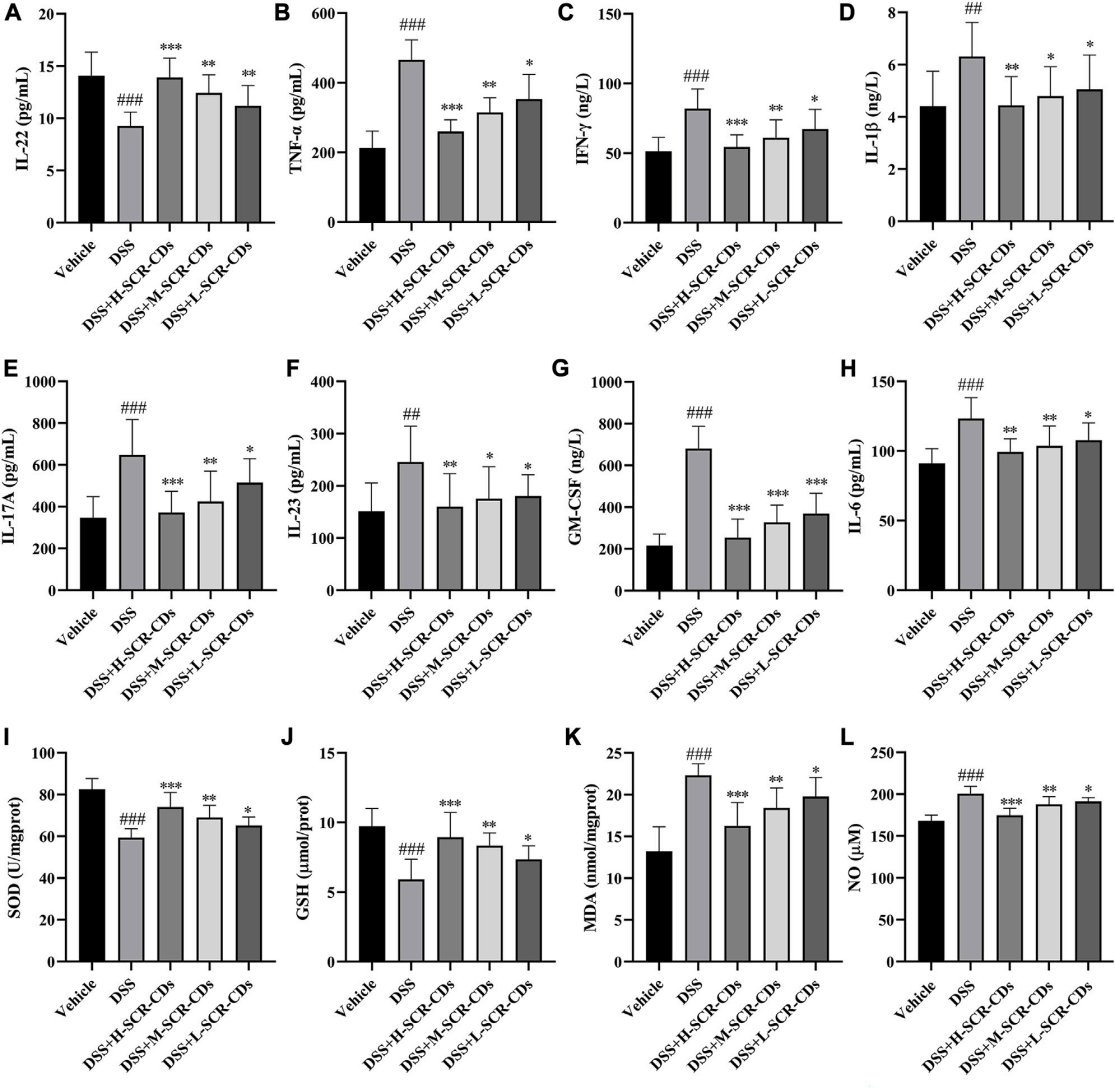
Effects of SCR-CDs on inflammatory cytokines and oxidative stress indicators associated with the mucosal microecological environment in DSS-induced mice. The detection of inflammatory cytokines IL-22 **(A)**, TNF-α **(B)**, IFN-γ **(C)**, IL-1β **(D)**, IL-17A **(E)**, IL-23 **(F)**, GM-CSF **(G)** and IL-6 **(H)**. The detection of oxidative stress indicators SOD **(I)**, GSH **(J)**, MDA **(K)** and NO **(L)**. Data are expressed as mean ± SD (*n* = 8). ^###^ *p* ˂ 0.001 versus vehicle group; *p ˂ 0.05, **p ˂ 0.01 and ***p ˂ 0.001 versus DSS group.

The disruption of the epithelial barrier and disassembly of intestinal TJ proteins facilitated the exposure to gut bacteria and subsequent pro-inflammatory stimulation (Figures 5A–H). The structural and functional disruption of the barrier in UC also led to the absence of IECs. To maintain intestinal homeostasis, further differentiation of IECs is necessary to restore the integrity of the mucosal barrier and epithelial function. The phase was facilitated by regulatory proteins, including IL-22, which represents one of the key cytokines regulated by IL-23 (Neurath and Travis, 2012). In addition to preserving the integrity of the enteric epithelium, IL-22 has the capacity to influence the composition of the microbiota and prevent bacterial translocation (Renga et al., 2022). This study, consistent with the literature reported, demonstrated that the levels of IL-22 were downregulated in DSS-induced colitis. Thus, it was conceivable that elevation of IL-22 and TJ levels after SCR-CDs treatment could maintain paracellular permeability of the intestinal epithelium and block the process of microbiota invasion in colonic tissue.

The infiltration of activated immune cells promotes the abnormal expression of relevant inflammatory cytokines during the perturbation of this mucosal barrier (Ban et al., 2022). IL-23 enhances the effector function of both innate and adaptive lymphocytes, leading to the production of IL-17A and GM-CSF. Furthermore, the IL-23/granulocyte-macrophage colony-stimulating factor (GM-CSF) axis plays a crucial role in driving neutrophil recruitment and exacerbating intestinal inflammation (Griseri et al., 2012). During the inflammatory response, monocytes migrate from the bloodstream into infected tissues and eventually differentiate into mature macrophages in the presence of GM-CSF. After activation, macrophages secrete pro-inflammatory cytokines such as TNF-α, IL-1β, and IL-6, which enhance leukocyte recruitment to the inflammatory foci. In comparison to the vehicle group, the DSS-treated group exhibited a significant increase in the levels of TNF-α, IFN-γ, IL-1β, IL-17A, IL-23, GM-CSF, and IL-6. However, each group treated with SCR-CDs exhibited a varying degree of mitigation of these changes. Collectively, these data indicate that SCR-CDs exert an anti-colitis effect, as evidenced by the reduction of inflammatory cell infiltration and the regulation of cytokines in DSS-induced mice.

The oxidative stress also accompanies the pathological processes involved in the occurrence and development of UC. SOD and GSH are indispensable antioxidant substances in the body, crucial for mitigating the generation of free radicals during intense inflammation (Duan et al., 2023). MDA is a product of lipid peroxidation in the body, and its quantification provides insight into the extent of lipid peroxidation (Huang J. Q. et al., 2022). Dysregulated expression of NO also contributes to elevated oxidative stress levels (Amirshahrokhi and Imani, 2023). A previous report has proposed that CDs can protect cells from oxidative stress. This protective mechanism is associated with intracellular ROS elimination and the intracellular SOD production (Xu et al., 2015). Consistently, the results of this study demonstrated that the activities of SOD and GSH in the DSS group were significantly lower than those in the vehicle group, while the content of MDA and NO was increased, indicating that the mice after DSS induction were experiencing peroxidative stress. However, compared to the DSS group, upon administration of SCR-CDs, the activities of SOD and GSH were increased, and the levels of MDA and NO were decreased, suggesting that SCR-CDs had a protective effect against oxidative stress injury in the UC model mice (Figure 5I, L).

According to the results, SCR-CDs exerted the anti-colitis effect as evidenced by preventing activation of the inflammatory cascade and inhibiting oxidative stress in DSS-induced mice, and thus the homeostasis of the intestinal microbiological environment.

### 3.5 SCR-CDs modulated the gut microbiota of DSS-induced mice

The intestinal microbial imbalance in IBD has been extensively reported, and this imbalance can exacerbate the inflammatory process. To elucidate the impact of SCR-CDs on the gut microbiota in DSS-induced mice, we conducted 16 S rDNA profiling. Additionally, we investigated the effect of SCR-CDs on the intestinal microbiome structure by analyzing the distribution of gut microbiota species and their relative abundance among the three groups.

**FIGURE 6:**
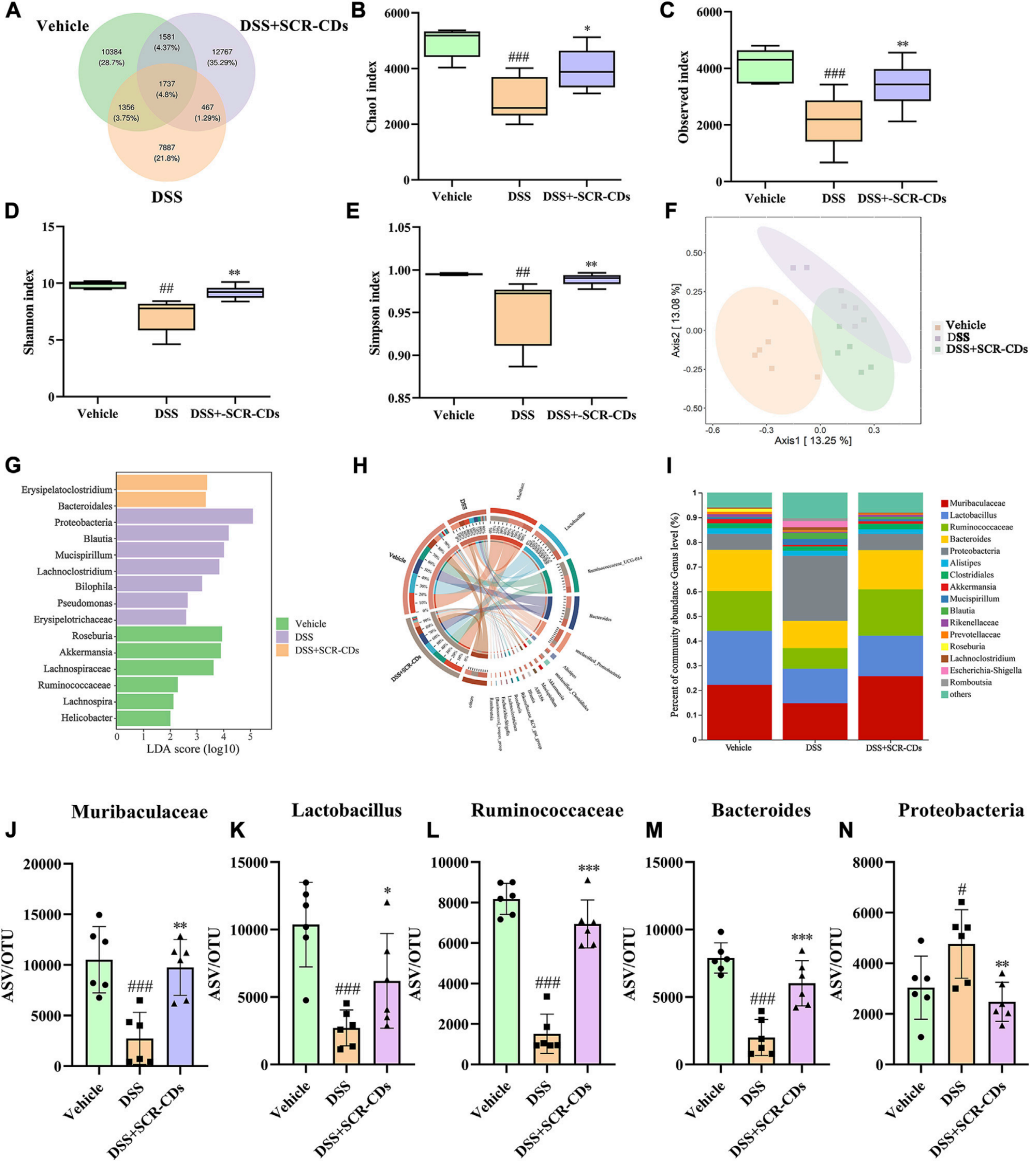
Regulatory effect of SCR-CDs on gut microbiota disturbance induced by DSS in colitis mice. **(A)** Venn diagram displaying common or unique species among the three groups. **(B–E)** Alpha diversity indexes were evaluated according to the OTU numbers of each group. ^##^ *p* ˂ 0.01, ^###^ *p* ˂ 0.001 versus vehicle group; *p ˂ 0.05, **p ˂ 0.01 and ***p ˂ 0.001 versus DSS group. **(F)** Multiple sample principal coordinates analysis (PCoA) on the bacterial flora in feces. **(G)** Differentially enriched gut microbiota in each group of mice at the genus level by linear discriminant analysis (LDA). LDA score higher than 2 indicates a higher relative abundance in the corresponding group than that in other groups. **(H)** Circos plot showing the distribution of genus level between the different treatment groups. **(I)** The column chart of the relative distribution of each group at the genus level. **(J-N)** Significantly different bacteria at the genus level in the fecal microbiota.

In the comparison between the vehicle group and the DSS group, 467 overlapping Operational Taxonomic Units (OTUs) were identified, representing 1.29% of the total OTUs. Similarly, the DSS group and the SCR-CDs group exhibited 1356 overlapping OTUs, accounting for 3.75% of the total OTUs. Notably, the DSS-treated mice exhibited a lower number of OTUs compared to the vehicle or SCR-CDs-treated mice (Figure 6A). As depicted in Figures 6B–E, the assessment of alpha diversity through the Chao1 index, Observed index, Shannon index, and Simpson index indicated a notable reduction in microbial richness and community diversity in the DSS-treated groups. However, the administration of SCR-CDs effectively restored the alpha diversity of gut microbiota. Moreover, based on OTU abundance, the principal coordinates analysis (PCoA) revealed that the microbiota of the SCR-CDs group exhibited greater similarity with that of the vehicle group, as compared to the DSS-treated group (Figure 6F). The above results indicate that the treatment with SCR-CDs could partially reverse the dysbiosis of gut microbiota in DSS-induced colitis mice.

Linear discriminant analysis effect size (LEfSe) identified the characteristic microorganisms within each group, and the taxonomic hierarchy of these characteristic microorganisms was shown in Figure 6G. Compared to the vehicle group, the DSS-induced colitis group exhibited an abundance of *Proteobacteria*, *Blautia*, and *Mucispirillum* in the intestine. After oral administration of SCR-CDs, an increase in beneficial bacteria, including *Bacteroidales*, was detected. To further investigate the relationship between SCR-CDs-regulated gut microbiota and UC, a significant change in abundance was analyzed. As shown in Figure 6H, N, the abundance of *Muribaculaceae, Lactobacillus, Ruminococcaceae,* and *Bacteroides* significantly decreased while the abundance of *Proteobacteria*, *Akkermansia*, and *Mucispirillum* significantly increased after the DSS treatment in genus level. However, after the administration of SCR-CDs, the overall gut microbiota distributions converged to be similar to those of the untreated group. These results indicated that SCR-CDs could help to restore the gut microbiota structure in mice with DSS-induced colitis.

Literature research has shown that intestinal epithelial damage and disruption results in the translocation of commensal bacteria in the bowel wall, which played a pivotal role in the pathogenesis of UC (Glassner et al., 2020). Typically, in healthy individuals, *Firmicutes* and *Bacteroidetes* constitute approximately 90% of the gut microbiota (Lee and Chang, 2021). The imbalance of the *Bacteroidetes*/*Firmicutes* ratio has been implicated in predisposition to IBD (Stojanov et al., 2020). Although the flora communities of individuals were not identical to those of animals, our study revealed that the DSS model exhibited intestinal injury accompanied by a decrease in microbiota abundance and an imbalance between probiotics and harmful bacteria. Our study observed marked increases in *Bacteroidetes* after oral administration of SCR-CDs. Previous researchers have demonstrated that *Lactobacillus* could suppress TNF-α expression, improved antioxidant capacity, and directly compete with pathogenic bacteria, thus contributing to ameliorated body weight loss (Hu et al., 2018; Chen et al., 2019). Remarkably, the administration of SCR-CDs in mice protected against the substantial depletion of beneficial microorganisms, such as *Lactobacillus*, *Ruminococcaceae*, and *Muribaculaceae* (Hao et al., 2021). Conversely, an increase in harmful bacteria, such as *Proteobacteria*, which contains many potential pathogens, has been proposed as a diagnostic marker for dysbiosis and an increased risk of disease in the colon (Shin et al., 2015). Therefore, we hypothesized that SCR-CDs could impact the abundance of dominant bacteria in the intestine. The main bacterial flora and associated pathological states were described in detail in Supplementary Table S1. Our study revealed that SCR-CDs increased the diversity of the intestinal microbiota and restored the balance between beneficial and pathogenic bacteria. These findings demonstrate that the protective effect of SCR-CDs on colitis might be attributed to its regulation of gut microbiota correlated with inflammation.

### 3.6 Biotoxicity analysis of SCR-CDs *in vitro* and *in vivo*

**FIGURE 7:**
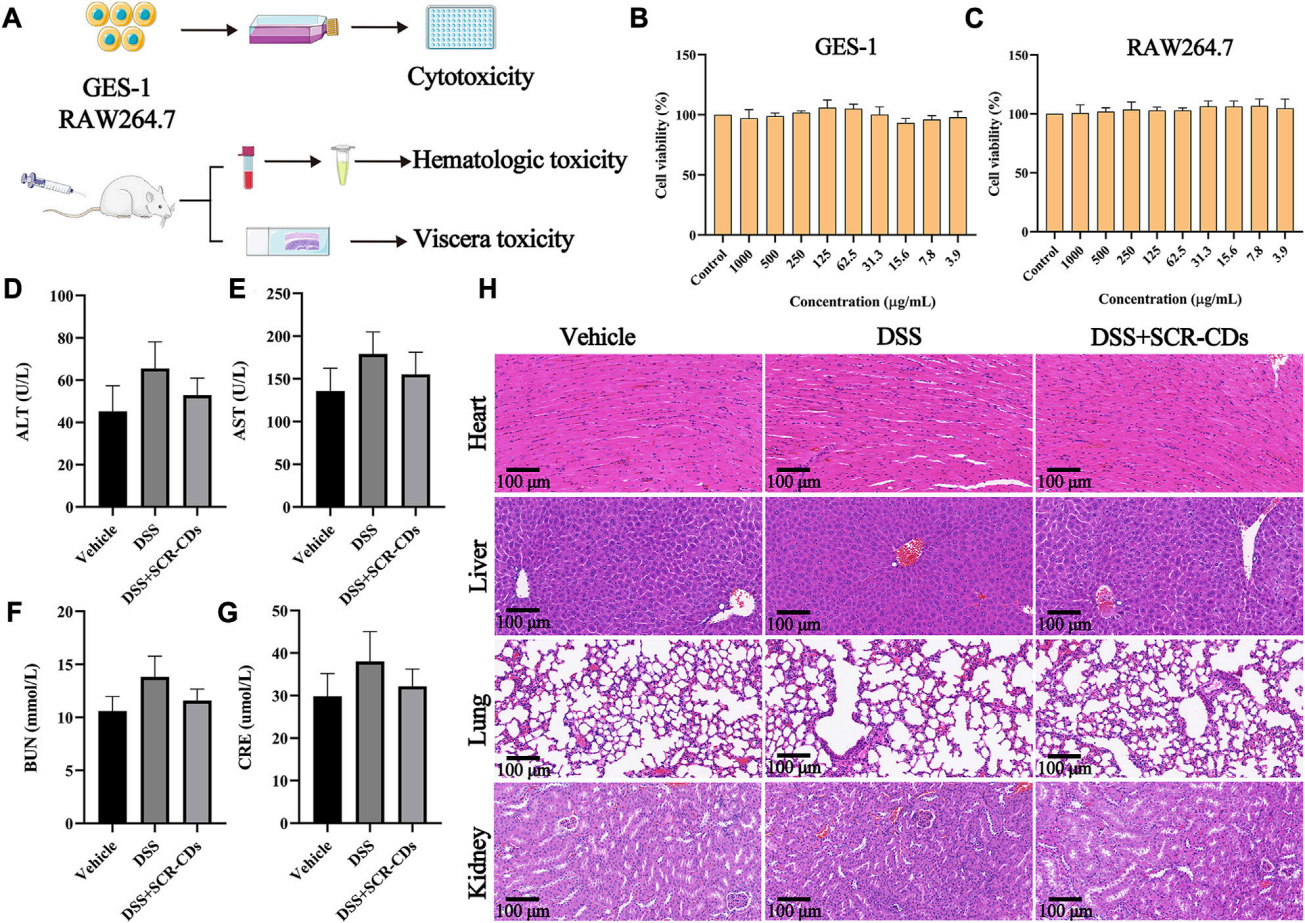
*In vitro* and *in vivo* biosafety evaluation of SCR-CDs. **(A)** Experimental scheme for toxicity detection. CCK-8 analysis was performed with GES-1 cells **(B)** and RAW264.7 cells **(C)** in various concentrations of SCR-CDs for 24 h. Blood was analyzed for ALT **(D)**, AST **(E)**, BUN **(F)** and CRE **(G)**. **(H)** Histological evaluation of four major organs of mice in different groups (×200 magnification, Scar bar = 100 μm). Data are expressed as mean ± SD (*n* = 8).

CDs had great potential for therapeutic applications with the majority of nanoparticles approved for healthcare applications being carbon-based (Truskewycz et al., 2022). Despite the publication of encouraging results regarding the suitability of CDs for biomedical applications, it is essential to evaluate their biosafety from various perspectives before including them in clinical studies (Rennick et al., 2021). Consequently, after the administration of SCR-CDs, we conducted the CCK-8 assay to assess its cytotoxic effect and observed changes in blood biochemical markers and histological sections of major organs (Figure 7A). At the cellular level, we investigated the cytotoxicity of SCR-CDs against GES-1 cells and RAW 264.7 cells at different dose concentrations. Interestingly, no significant effect on the survival rates of GES-1 cells and RAW 264.7 cells was observed under the present experimental conditions (Figure 7B, C). Moreover, the viabilities of GES-1 cells and RAW 264.7 cells remained above 90% across a wide dosing range (ranging from 3.9 to 1,000 μg/mL), indicating that SCR-CDs had no adverse impact on cell growth. It is worth mentioning that SCR-CDs showed no toxicity towards GES-1 cells, providing safety data regarding the oral administration of SCR-CDs.

Satisfactorily, blood chemical examinations, including ALT, AST, BUN, and CRE levels, in mice orally administered with SCR-CDs, showed no noticeable abnormalities (Figures 7D–G). Furthermore, we conducted a evaluation of potential toxicity towards multiple organs *in vivo*. Histological analysis and H&E staining demonstrated that no morphological or pathological abnormalities were detected in any of the treatment groups, indicating that SCR-CDs intervention had little impact on the major organs of mice (Figure 7H, Supplementary Figure S4). Based on the results, SCR-CDs exhibited excellent biocompatibility and biosafety in both *in vitro* and *in vivo* settings.

## 4 Conclusion

In this study, we conducted a comprehensive characterization of the morphological structure and functional groups of SCR-CDs, which were isolated from SCR. Moreover, we evaluated the protective effect of SCR-CDs against UC using a widely accepted DSS-induced disease model in mice. The hygroscopic capacity and hemostatic bioactivity displayed by SCR-CDs were found to be beneficial in ameliorating the main manifestations of UC, particularly bloody diarrhea. Furthermore, the treatment with SCR-CDs led to an improvement in the intestinal microecological environment by restoring the gut barrier and regulating the microflora, thereby modulating the hyperimmune status. This study offers novel insights into the carbonization process of pure plants in the preparation of carbon dots. We believe that the environmentally friendly, cost-effective, and safer SCR-CDs hold great potential to augment therapeutic strategies for UC.

## Data Availability

The original contributions presented in the study are included in the article/Supplementary Material, further inquiries can be directed to the corresponding authors.
