# Supplementary material for: Protective effect of carbon dots derived from scrambled Coptidis Rhizoma against ulcerative colitis in mice
Source: Front Mol Biosci. 2023 Aug 30;10:1253195. doi: 10.3389/fmolb.2023.1253195 (PMC10498776; doi:10.3389/fmolb.2023.1253195)
Supplement: Supplementary file 1 [file Table1.DOCX]

Supplementary Material

# Supplementary Figures

**
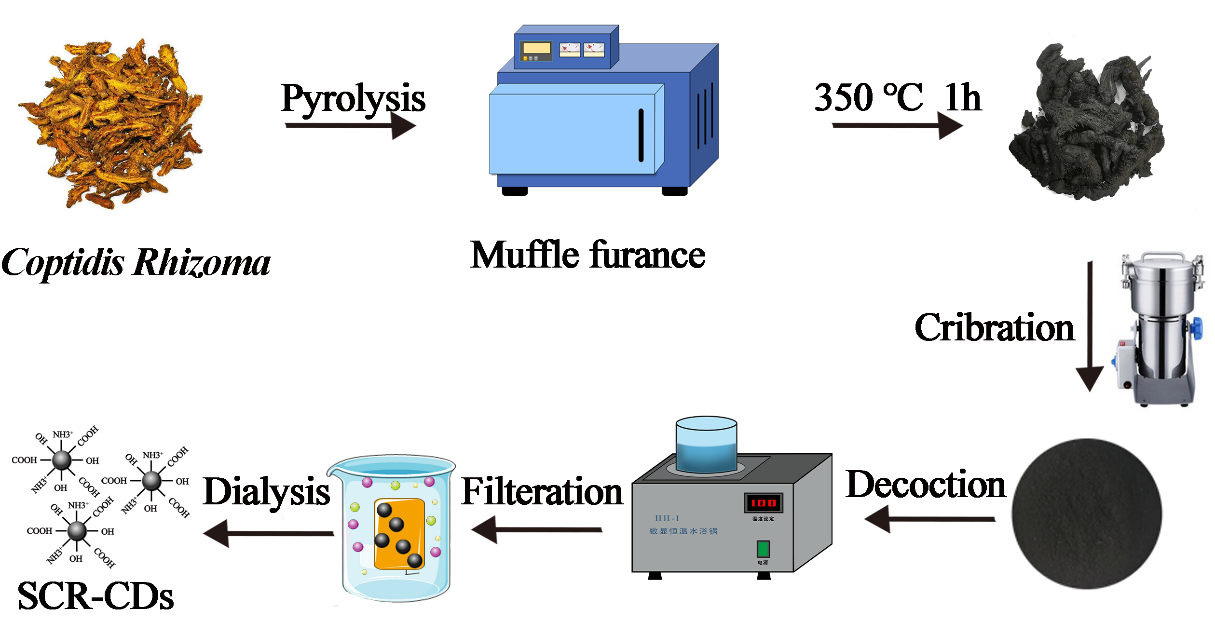
**

**Supplementary Figure S1.** Flowchart of synthesis of SCR-CDs.

**
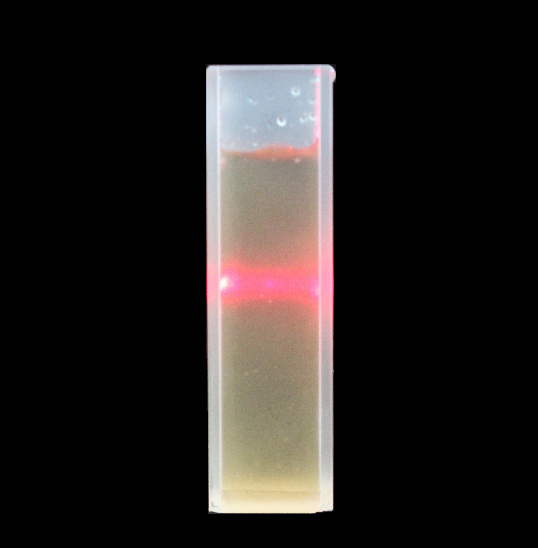
**

**Supplementary Figure S2.** Tyndall effect of liquid containing SCR-CDs. Illumination with infrared visible light and appearation of the micelles in the liquid.

**
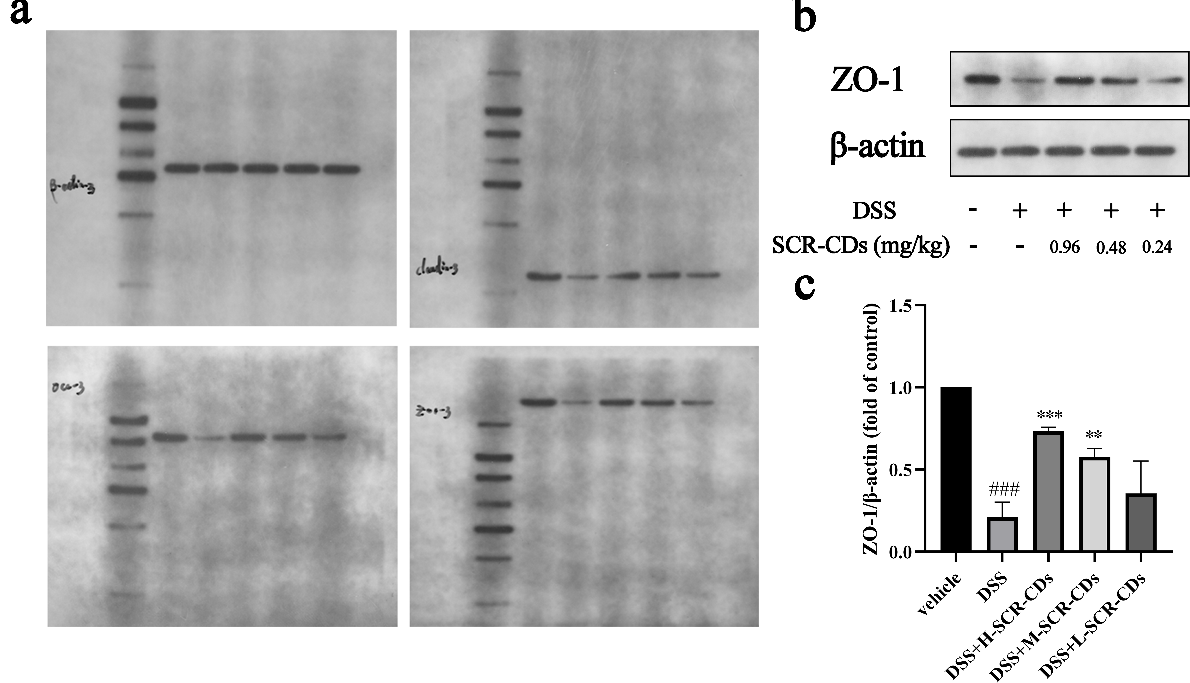
**

**Supplementary Figure S3.** **(a)** Raw data for western blot. **(b)** Immunoblot for ZO-1 in colonic tissue. **(c)** The densitometric analysis of ZO-1 immunoblot in each group.

**
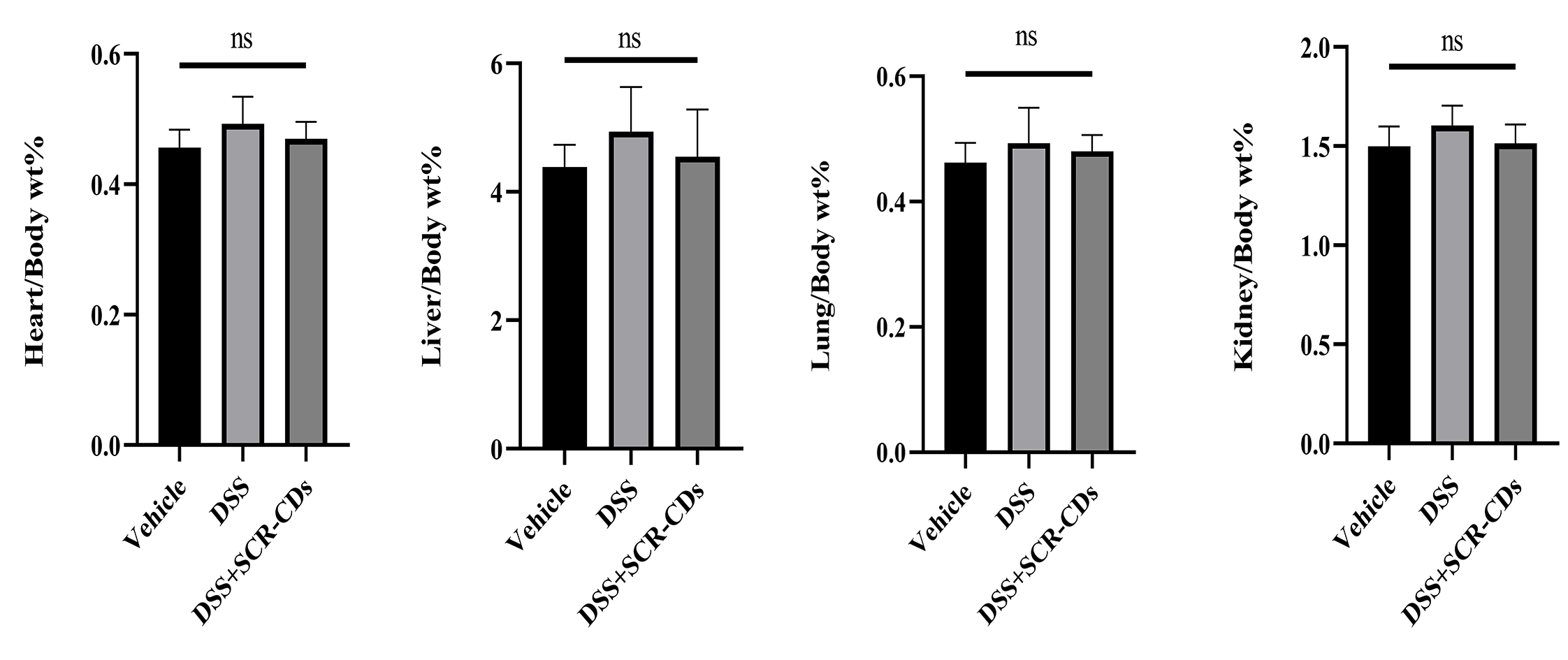
**

**Supplementary Figure S4.** Organ weight of mice in different groups. ns, not statistically significant.

1. **Supplementary Table**

**Supplementary Table S1.** The main bacterial flora and associated pathological states.

| **Main bacterial flora at genus level** | **Beneficial or harmful bacteria** | **Associated with pathological states** |
| --- | --- | --- |
| Muribaculaceae | Beneficial | Inhibit the CD8^+^T cell activation to tolerate the immunity stimulation and present a negative correlation with inflammation status (Shang et al., 2021). |
| Ruminococcaceae | Benefical | Enhance intestinal function by increasing mucin secretion and the enhancement of tight-junctions; Inhibit the signaling pathways of pro-inflammatory cytokines (Morgan et al., 2012). |
| Lactobacillus | Beneficial | Inhibit harmful bacteria and improve gastrointestinal barrier function (Azad et al., 2018). |
| Bacteroides | Harmful | Increase colonic inflammation in UC and modulate inflammatory response through regulating Treg cells response (Cui et al., 2021; Zhao et al., 2019). |
| Proteobacteria | Harmful | Promote the production of excessive pro-inflammatory cytokines (Qu et al., 2021). |
| Clostridiales | Harmful | Contribute to perturbations in the immune function of lamina propria cell (Clemente et al., 2018). |
| Alistipes | Beneficial | Protect intestinal barrier function, block intestinal inflammation and oxidative stress injury (He et al., 2022); Negatively correlated with the disease symptoms and colon inflammation (Wu et al., 2019). |
| Akkermansia | Beneficial | Anti-inflammatory effect by improving neutrophil infiltration (Liu et al., 2021); reduce intestinal permeability and increase the thickness of the mucus layer (Anhê et al., 2015). |
| Blautia | Beneficial | Produce butyrate which have anti-inflammatory activities, protect against pathogen invasion, modulate the immune system (Miquel et al., 2013) . |
| Ruminiclostridium | Beneficial | Degrade polysaccharides to produce acetate and butyrate and promote the development of the immune system (Zou et al., 2020). |
| Escherchia/Shigella | Harmful | Adhered to the mucosal epithelial cells of the colon was positively correlated with pro-inflammatory cytokines and destroy the integrity of the intestinal barrier (Jia et al., 2018). |

# Supplementary references

Shang, L., Liu, H., Yu, H., Chen, M., Yang, T., Zeng, X., et al. (2021). Core Altered Microorganisms in Colitis Mouse Model: A Comprehensive Time-Point and Fecal Microbiota Transplantation Analysis. *Antibiotics (Basel)* 10 (6), 643. doi: 10.3390/antibiotics 10060643.

Morgan, X. C., Tickle, T. L., Sokol, H., Gevers, D., Devaney, K. L., Ward, D. V., et al. (2012). Dysfunction of the intestinal microbiome in inflammatory bowel disease and treatment. *Genome Biol* 13 (9), R79. doi: 10.1186/gb-2012-13-9-r79.

Azad, M. A. K., Sarker, M., Li, T.,Yin, J. (2018). Probiotic Species in the Modulation of Gut Microbiota: An Overview. *Biomed Res Int* 2018, 9478630. doi: 10.1155/2018/9478630.

Cui, L., Guan, X., Ding, W., Luo, Y., Wang, W., Bu, W., et al. (2021). Scutellaria baicalensis Georgi polysaccharide ameliorates DSS-induced ulcerative colitis by improving intestinal barrier function and modulating gut microbiota. *Int J Biol Macromol* 166, 1035-1045. doi: 10.1016/j.ijbiomac.2020.10.259.

Zhao, H., Cheng, N., Zhou, W., Chen, S., Wang, Q., Gao, H., et al. (2019). Honey Polyphenols Ameliorate DSS-Induced Ulcerative Colitis via Modulating Gut Microbiota in Rats. *Mol Nutr Food Res* 63 (23), e1900638. doi: 10.1002/mnfr.201900638.

Qu, Y., Li, X., Xu, F., Zhao, S., Wu, X., Wang, Y., et al. (2021). Kaempferol Alleviates Murine Experimental Colitis by Restoring Gut Microbiota and Inhibiting the LPS-TLR4-NF-κB Axis. *Front Immunol* 12, 679897. doi: 10.3389/fimmu.2021.679897.

Clemente, J. C., Manasson, J., Scher, J. U., (2018). The role of the gut microbiome in systemic inflammatory disease. *Bmj* 360, j5145. doi: 10.1136/bmj.j5145.

He, X. Q., Liu, D., Liu, H. Y., Wu, D. T., Li, H. B., Zhang, X. S., et al. (2022). Prevention of Ulcerative Colitis in Mice by Sweet Tea (Lithocarpus litseifolius) via the Regulation of Gut Microbiota and Butyric-Acid-Mediated Anti-Inflammatory Signaling. *Nutrients* 14 (11), 2208. doi: 10.3390/nu14112208.

Wu, M., Li, P., An, Y., Ren, J., Yan, D., Cui, J., et al. (2019). Phloretin ameliorates dextran sulfate sodium-induced ulcerative colitis in mice by regulating the gut microbiota. *Pharmacol Res* 150, 104489. doi: 10.1016/j.phrs.2019.104489.

Liu, J. H., Chen, C. Y., Liu, Z. Z., Luo, Z. W., Rao, S. S., Jin, L., et al. (2021). Extracellular Vesicles from Child Gut Microbiota Enter into Bone to Preserve Bone Mass and Strength. *Adv Sci (Weinh)* 8 (9), 2004831. doi: 10.1002/advs.202004831.

Anhê, F. F., Roy, D., Pilon, G., Dudonné, S., Matamoros, S., Varin, T. V., et al. (2015). A polyphenol-rich cranberry extract protects from diet-induced obesity, insulin resistance and intestinal inflammation in association with increased Akkermansia spp. population in the gut microbiota of mice. *Gut* 64 (6), 872-883. doi: 10.1136/gutjnl-2014-307142.

Miquel, S., Martín, R., Rossi, O., Bermúdez-Humarán, L. G., Chatel, J. M., Sokol, H., et al. (2013). Faecalibacterium prausnitzii and human intestinal health. *Curr Opin Microbiol* 16 (3), 255-261. doi: 10.1016/j.mib.2013.06.003.

Zou, J., Shen, Y., Chen, M., Zhang, Z., Xiao, S., Liu, C., et al. (2020). Lizhong decoction ameliorates ulcerative colitis in mice via modulating gut microbiota and its metabolites. *Appl Microbiol Biotechnol* 104 (13), 5999-6012. doi: 10.1007/s00253-020-10665-1.

Jia, W., Xie, G.,Jia, W., (2018). Bile acid-microbiota crosstalk in gastrointestinal inflammation and carcinogenesis. *Nat Rev Gastroenterol Hepatol* 15 (2), 111-128. doi: 10.1038/nrgastro.2017.119.
